# Emotional Touch Nursing Competencies Model of the Fourth Industrial Revolution: Instrument Validation Study

**DOI:** 10.2196/67928

**Published:** 2024-12-16

**Authors:** Sun-Young Jung, Ji-Hyeon Lee

**Affiliations:** 1 Research Institute of Nursing Science College of Nursing Daegu Catholic University Daegu Republic of Korea; 2 College of Nursing Daegu University Daegu Republic of Korea

**Keywords:** nurse, therapeutic touch, clinical competence, factor analysis, statistical, reliability, scale, tool, nursing, industrial revolution, competencies, health care, emotional, interview, collaborative practice, learning agility, professional commitment, positive self-worth, compliance, ethics, practice ability, relationship ability, nursing sensitivity

## Abstract

**Background:**

The Fourth Industrial Revolution is transforming the health care sector through advanced technologies such as artificial intelligence, the Internet of Things, and big data, leading to new expectations for rapid and accurate treatment. While the integration of technology in nursing tasks is on the rise, there remains a critical need to balance technological efficiency with empathy and emotional connection. This study aims to develop and validate a competency model for emotional touch nursing that responds to the evolving demands of the changing health care environment.

**Objective:**

The aims of our study are to develop an emotional touch nursing competencies model and to verify its reliability and validity.

**Methods:**

A conceptual framework and construct factors were developed based on an extensive literature review and in-depth interviews with nurses. The potential competencies were confirmed by 20 experts, and preliminary questions were prepared. The final version of the scale was verified through exploratory factor analysis (n=255) and confirmatory factor analysis (n=256) to assess its validity and reliability.

**Results:**

From the exploratory analysis, 8 factors and 38 items (client-centered collaborative practice, learning agility for nursing, nursing professional commitment, positive self-worth, compliance with ethics and roles, nursing practice competence, nurse-client relationship, and nursing sensitivity) were extracted. These items were verified through convergent and discriminant validity testing. The internal consistency reliability was acceptable (Cronbach α=0.95).

**Conclusions:**

The findings from this study confirmed that this scale has sufficient validity and reliability to measure emotional touch nursing competencies. It is expected to be used to build a knowledge and educational system for emotional touch nursing.

## Introduction

The Fourth Industrial Revolution, characterized by the technological convergence of existing and new data through the blurring of physical and digital boundaries, is driving exponential changes across various sectors at an unprecedented pace [1]. Anticipated transformations and revolutions in the economic, social, and cultural domains are enabled by the convergence of advanced technologies such as artificial intelligence (AI), the Internet of Things, cloud computing, and big data [2]. At the core of these changes lie the health care sector, big data, information and communications technologies, and IT devices, facilitating precision diagnosis, telemedicine, and remote monitoring in medical settings [3].

With the advancement of scientific technology, health care recipients now expect rapid and accurate treatment through precise evidence-based medicine, as well as continuous disease management and health maintenance using IT technologies [3,4]. The medical field has evolved beyond uHealth, with diagnostics and certain support for the execution of nursing tasks increasingly being replaced by AI. Additionally, nursing models that use virtual reality for drug administration, as well as nursing robots that assist with tasks like meal delivery and medication, have been developed, leading to improved nursing quality [5]. Moreover, nurses are expected to analyze big data, provide diagnostic interventions tailored to individual needs according to algorithms, and deliver optimal nursing services with the assistance of AI [6]. Thus, the nursing field as a whole anticipates that the use of new technologies will enhance the efficiency of nursing tasks, alleviate work burdens, and pioneer independent activity areas [4].

However, alongside the rapid technological advancements, there is a growing emphasis on the humanistic and emotional aspects of nursing care, highlighting the importance of balancing the scientific and technological aspects of medicine. As nursing service recipients experience dehumanization in the face of digital transformation [4], there is a demand for more humanistic and delicate care. The increasing need for high-tech solutions like big data, AI, and robots underscores the importance of empathy and human touch—areas where nursing uniquely excels in understanding and connecting emotionally with patients [4,7]. In this context, emotional touch nursing can be described as a professional nursing activity that delivers integrated care through direct physical contact and nonverbal communication, using various resources and collaborating with other health care professionals to sensitively address the health problems of nursing recipients [7].

In recent years, there has been a growing consensus on the importance of adapting nursing to the Fourth Industrial Revolution era, with continuous research being conducted on emotional intelligence, person-centered care, and therapeutic touch [8-11]. Emotional intelligence, widely used in Korean nursing, was originally developed for business students and later adapted to South Korea to reflect the sociocultural characteristics of the country. Although its validity and reliability were verified, it was adapted for nursing students, not nurses, and consists of only 4 factors with 4 items each, making it insufficient in terms of the number of items for each subdomain. Furthermore, it was found to be influenced by personal traits such as personality characteristics [8]. Person-centered care tools include the Person-Centered Care Assessment Tool (P-CAT), developed overseas and translated into Korean [9], and tools developed to emphasize specific aspects such as intensive care unit nursing practice, family involvement, and end-of-life care [10], which pose challenges in measuring emotional touch nursing that moves recipients in the domestic, social, and cultural contexts [7]. Therefore, it is necessary to prioritize the development of tools that reflect the concept of emotional touch competency, suitable for the rapidly changing nursing environment in the Fourth Industrial Revolution era.

Competency modeling can be used as a method for constructing a model of nurses’ emotional touch nursing competencies. This approach involves developing a competency model that includes complex elements, such as knowledge, skills, personality, attitudes, problem-solving abilities, and interpersonal skills, and visually represents the relationships between these competencies as a multidimensional concept map [12]. The development of competency models through competency modeling can be effectively used in the development of educational programs as it provides specific skills or behaviors that can be developed through education [13]. Competency-based approaches have been reported to be more effective than other approaches in terms of positive psychology and reinforcement [14], with effectiveness verified in competency-based educational programs across various fields [15-18]. In particular, previous studies on nurse competencies emphasize the use of competency models to develop competent and skilled nursing personnel [19], highlighting the need for further research to identify and analyze nurses’ competencies from diverse and in-depth perspectives in clinical settings [20]. Therefore, this study aims to construct a model of emotional touch nursing competencies in response to diverse nursing needs in the rapidly changing Fourth Industrial Revolution era, using a shortened competency modeling method based on expert panels commonly used among expert groups [21]. The specific objectives are mentioned in the next sections.

## Methods

### Study Design

This methodological study used a competency modeling approach to create a model of emotional touch nursing competencies within the context of the Fourth Industrial Revolution.

### Competency Model Construction Process

#### Phase 1: Construction of Potential Competency Model

##### Emotional Touch Nursing Competencies From a Conceptual Framework

To develop an emotional touch nursing competencies model through competency modeling for nurses, the components of the scale were identified based on a literature review and prior studies on the concept analysis of emotional touch through interviews with nurses and nursing recipients [7]. The identified competencies for emotional touch nursing include a total of 12 competencies: professional nursing skills, nursing sensitivity, interpersonal skills, leadership, educational competency, spirituality, resilience, judgment, learning sensitivity, IT use skills, collaboration with other health care professionals, ethical sensitivity, and humanistic recalibration.

##### Delphi Survey Using Expert Panel

A Delphi survey was conducted with the same expert group on the 12 emotional touch nursing competencies identified from prior studies [7]. A shortened competency modeling method [21] was used, which requires securing more than 20 experts in the field. The expert group consisted of 20 members—5 clinical nurses, 5 clinicians, 8 nurse educators, and 2 professors of medical humanities and bioethics. The survey period was from February 1, 2021, to April 30, 2021. In the first Delphi round, experts were asked to provide opinions on additional or deleted items using an open-ended questionnaire based on the competencies identified in the prior study. The first round results indicated the need to expand the conceptual scope to include “patient-centered care” as a new competency. Consequently, “patient-centered care” was added, bringing the total to 13 competencies for the second Delphi round, where experts were asked to evaluate the appropriateness of the definitions and names of the competencies using an open-ended questionnaire. The second round results confirmed that the 13 competencies were evenly distributed among the attributes of knowledge, skills, self-concept, and traits. In the third Delphi round, the appropriateness of the competencies was confirmed using a 5-point Likert scale, with all competencies scoring 4 or above, ensuring expert validity. Thus, a potential competency model was constructed consisting of patient-centered care, professional nursing skills, nursing sensitivity, interpersonal skills, leadership, educational competency, spirituality, resilience, judgment, learning sensitivity, IT use skills, collaboration with other health care professionals, ethical sensitivity, and humanistic recalibration.

#### Phase 2: Validation and Finalization of the Competency Model

##### Development of Preliminary Items

Based on the potential competency model construction process, 13 competencies were finalized, and the first set of preliminary items was developed to measure emotional touch nursing competencies. The preliminary items included 11 items on patient-centered care, 15 on professional nursing skills, 12 on nursing sensitivity, 13 on interpersonal skills, 14 on leadership, 15 on educational competency, 17 on spirituality, 22 on resilience, 11 on judgment, 11 on learning sensitivity, 12 on IT use skills, 7 on collaboration with other health care professionals, and 16 on ethical sensitivity and humanistic recalibration, totaling 176 items. The scale was a 5-point Likert scale, with higher scores indicating higher emotional touch nursing competencies.

##### Content Validity Testing

The initial set of preliminary items underwent content validity testing by the expert group of 20 members for competency modeling. The content validity of the preliminary items and the appropriateness of each competency attribute were assessed using a 4-point Likert scale, where 1 indicated “not at all appropriate” and 4 indicated “very appropriate.” Open-ended questionnaires were also used to collect opinions on any necessary revisions, additions, or deletions of items. The content validity testing results, with an Item-Content Validity Index (I-CVI) criterion of 0.78 or higher, led to the identification of 51 items. The second set of preliminary items was structured as a questionnaire, with 2 researchers cross-examining the appropriateness of the items’ meaning, language, duplication, or ambiguity, and their relevance to the subfactors of the conceptual framework. A Korean language expert and an education expert reviewed the items, refining the preliminary scale to a final selection of 51 items.

### Participants

The study participants were nurses with more than 3 months of experience working in secondary or tertiary hospitals located in the Seoul, Daegu, and Gyeongbuk regions who understood the aim of the study and voluntarily agreed to participate. Considering that the sample size for factor analysis should be 4 to 5 times the number of items developed, and the ideal sample size for item analysis is 2 to 10 times the number of questions [22], in this study, 546 nurses were targeted, accounting for the number of items and potential dropouts. A total of 511 valid responses were obtained after excluding 25 insufficient responses and 10 nonresponses, with 255 used for exploratory factor analysis (EFA) and 256 for confirmatory factor analysis (CFA).

### Research Tools

To verify the criterion validity of the preliminary scale, the P-CAT was used. This tool consists of 25 items across 5 factors (7 items for relationship, 4 for totality, 5 for respect, 5 for individualization, and 4 for empowerment) measured on a 5-point Likert scale, with higher scores indicating a higher level of person-centered care. The reliability at the time of development was Cronbach α=0.94, and in this study, the reliability was Cronbach α=0.97.

### Ethical Considerations

Data collection was conducted from May 1 to May 31, 2021, after obtaining approval from the Daegu Catholic University Institutional Review Board (CUIRB-2020-0011). The aims of the study, data collection methods, confidentiality of the data, and participants’ rights to withdraw from the study were explained to the participants. Participants were provided with a written consent form, which they could read thoroughly before voluntarily agreeing to participate in the study. Data were stored on a password-protected computer, accessible only to the researchers, and the data will be kept for 3 years after the study’s conclusion before being destroyed. Participants were given a small gift of 5000 KRW (equivalent to US $4.42) as a token of appreciation for their participation.

### Data Analysis

Collected data were analyzed using SPSS Statistics (version 23.0; IBM Corp) and AMOS (version 24.0; IBM Corp) programs. First, the general characteristics of the study participants were analyzed using descriptive statistics, including frequency, percentage, mean, and SD. Second, construct validity was assessed through item analysis, EFA, CFA, and convergent and discriminant validity analysis. Item analysis involved checking the mean, SD, skewness, and kurtosis of each item to confirm normal distribution, as well as calculating item-total correlation and the reliability coefficient when removing specific items. Factor analysis suitability was tested using the Kaiser-Meyer-Olkin (KMO) and Bartlett test of sphericity. EFA used principal component analysis with varimax rotation to extract factors, while CFA evaluated the model fit using indices such as chi-square, chi-square/*df*, root mean square error of approximation (RMSEA), squared root mean-squared residual (SRMR), the goodness of fit index (GFI), and comparative fit index (CFI). Convergent validity was confirmed by examining standardized regression coefficients, construct reliability, and average variance extracted (AVE). Discriminant validity was verified by ensuring that the square of the correlation coefficients was smaller than the AVE. Third, criterion validity was measured by calculating Pearson correlation coefficients using person-centered care as the criterion variable. Fourth, reliability was assessed using the Cronbach α coefficient to determine internal consistency.

## Results

### General Characteristics

The average age of the participants was 34.4 years, with 287 (56.2%) out of 511 participants being unmarried. The majority had a bachelor’s degree in nursing, accounting for 384 (75.1%) participants. The average total clinical experience was 9.27 years. Regarding income, 211 (41.3%) participants earned between 2.5 million and 3 million KRW (US $1=1130.50 KRW, effective as of May 31, 2021) per month. Most participants worked in general wards (n=285, 55.8%), and the majority held the position of nurse (n=426, 83.4%; Table 1).

**Table 1:** General characteristics of participants (n=511).

Characteristics and categories			Values
**Age (years), mean (SD)**			34.4 (9.90)
**Marital status, n (%)**			
	Single	287 (56.2)	
	Married	221 (43.2)	
	Others	3 (0.6)	
**Education, n (%)**			
	Diploma	73 (14.3)	
	Bachelor’s degree	384 (75.1)	
	Master’s degree	53 (10.4)	
	Doctoral degree	1 (0.2)	
Total clinical career (year), mean (SD)			9.27 (7.53)
**Income (KRW^a^), n (%)**			
	<1,500,000	4 (0.8)	
	1,500,000 to >2,000,000	7 (1.4)	
	2,000,000 to >2,500,000	85 (16.6)	
	2,500,000 to >3,000,000	211 (41.2)	
	3,000,000 to >3,500,000	96 (18.8)	
	3,500,000 to >4,000,000	55 (10.8)	
	≥4,000,000	53 (10.4)	
**Working unit, n (%)**			
	General unit	285 (55.8)	
	Special unit	199 (38.9)	
	Outpatient clinic	27 (5.3)	
**Current position, n (%)**			
	Staff nurse	426 (83.3)	
	Charge nurse	56 (11.0)	
	Head nurse (unit manager)	23 (4.5)	
	Nursing team leader or more	6 (1.2)	

^a^US $1=1130.50 KRW, effective as of May 31, 2021.

### Item Analysis

In the item analysis conducted for the EFA of the preliminary items, the corrected item-total correlation for each item and the total set of items was examined. The correlation coefficient for item 6, “I believe that most events happen for a reason,” was found to be below 0.30. Items with a correlation coefficient above 0.80 may indicate redundancy with other items, while those below 0.30 are considered to contribute less to the tool [22]. Therefore, item 6 was deleted. The correlation coefficients of the remaining 50 items ranged from 0.44 to 0.67, and the Cronbach α value after item removal was 0.96.

### Exploratory Factor Analysis

#### Overview

To determine whether the 50 preliminary items identified in the item analysis were suitable for factor analysis, the KMO test was performed, resulting in a score of 0.93. The Bartlett test of sphericity yielded a value of 7304.34 (degrees of freedom=1176; *P*<.001), confirming that the data were appropriate for factor analysis. The factor analysis extracted 8 factors with eigenvalues greater than 1.0. The scree plot also showed a horizontal change in eigenvalues after the ninth factor, with the cumulative variance explained being 60.4%. Using varimax rotation, items with a factor loading of 0.40 or higher were considered significant, while those with a loading of 0.50 or higher were deemed highly significant [22]. As a result, 11 items with factor loadings below 0.40 and 1 item deemed to have a different attribute by 2 researchers were excluded, leaving 38 items. The communalities of each item ranged from 0.40 to 0.78, meeting the criteria. The factor loadings for the 38 items across 8 factors ranged from 0.45 to 0.82. The factors were composed of 3 to 11 items each, satisfying the basic assumption that each factor should include at least 3 items [22] (Table 2).

**Table 2:** Result of exploratory factor analysis.

Factors (number of items) and items			Factor loading		Eigenvalues		Accumulative variance, %
**F1^a^ (11)**					17.79		12.1
	24. I can lead the patient and family toward active participation in nursing.	0.72					
	23. I can apply nonverbal communication (therapeutic touch, positive face, eye gaze, etc) for client centered nursing depending on the individual characteristics.	0.62					
	25. I can provide an emotionally comfortable and cozy environment (noise pollution, eliminate odor, adjusting lighting brightness, etc) to the client.	0.60					
	35. I can determine priorities for educational needs, goals, and schedules through mutual discussions between the nurse and the client.	0.59					
	26. I can discuss with the patient, family, and colleagues about methods to provide client centered nursing.	0.57					
	37. I can provide constructive feedback by rechecking educational content after client education is completed.	0.55					
	32. I can provide an individual nursing education depending on the client’s needs, symptoms, characteristics, learning types (learning preferences or method), and self-care level.	0.52					
	34. I can help the client build a positive relationship with other patients, medical personnel, and others through appropriate communication.	0.47					
	33. I can provide client with education on available materials and human resources to help them adapt to the hospital environment.	0.46					
	45. I can discuss with colleagues and expertise groups (hospital ethics committee, etc) about the ethical issues that could arise when providing best nursing.	0.45					
	28. I can communicate with client to help them honest express emotions, thoughts, and opinions.	0.45					
**F2^b^ (4)**					2.55		20.5
	49. I can search and evaluate reliable internet-based material (clinical nursing guidelines, related papers, etc) to provide evidence-based practice.	0.81					
	48. I can use application programs (Excel, Power Point, Hangul, Zoom, YouTube, etc) to carry out whatever work I want to do.	0.80					
	50. I can use information and communication technologies (health care applications and patient education related videos, etc) to educate clients on effective health management.	0.73					
	36. I can educate the client’s using various methods such as videos, pamphlets, and role-play to elevate the effectiveness of education.	0.55					
**F3^c^ (4)**					2.12		28.2
	13. I feel self-esteem and a sense of vocation within the job.	0.82					
	12. I sense self-realization and value of life when I provide nursing to the client.	0.79					
	11. I believe nursing has a profound meaning in my life.	0.77					
	9. I feel pleased when I am learning or self-development in relation to my job.	0.54					
**F4^d^ (5)**					1.83		35.3
	1. I can overcome new challenges and adversities well, based on my past experiences and hope for the future.	0.72					
	3. I am a person who is needed and important to others.	0.69					
	4. I can set my life goals and grow for my own happiness.	0.68					
	2. I can make efforts to resolve any issues to achieve my goals.	0.65					
	5. When there is a challenging problem, I am able to find positive aspects that could arise in the process of resolving the problem.	0.52					
**F5^e^ (5)**					1.50		42.2
	43. I believe the act of caring for the client should be prioritized under any circumstances.	0.71					
	47. I believe it takes efforts and time to effectively use the hospital information system necessary for providing clinical or nursing services to the client.	0.62					
	44. I believe medical personnel should feel responsible if they or other colleagues are committing unethical medical practices, and take further action accordingly.	0.62					
	46. I believe good nursing involves equally respecting the human rights and self-determination rights of all individual clients.	0.54					
	31. I believe it is the responsibility of a nurse who performs a professional role to sensitively respond to new nursing knowledge and skills or techniques.	0.52					
**F6^f^ (3)**					1.45		48.6
	21. I can record the treatment and nursing intervention applied to the client pursuant to the nursing record guidelines.	0.80					
	22. I can use necessary emergency medications, medical devices, equipment, and so forth for nursing the patient and manage facilities.	0.79					
	20. I can provide expert nursing skills (core nursing skills including nutrition, elimination, medication, blood transfusion, etc) depending on the characteristics of the patient.	0.76					
**F7^f^ (3)**					1.22		55.1
	16. When the client expresses negative or positive thoughts and feelings toward me, I can acknowledge such thoughts and feelings.	0.75					
	15. When there is a discord between me and the client, I can constructively communicate and convey feelings and thoughts of either party.	0.70					
	17. I can exhibit a friendly attitude and maintain good relationships with patients.	0.63					
**F8^g^ (3)**					1.15		60.4
	27. I believe it is important to determine the treatment method preferred by the client within a possible scope.	0.65					
	30. I believe desirable nursing involves promptly responding to the vulnerable situations and needs of the patient.	0.59					
	29. I fully understand what impacts specific behavior of the nurse has on the client.	0.58					

^a^F1: client-centered collaborative practice.

^b^F2: learning agility for nursing.

^c^F3: nursing professional commitment.

^d^F4: positive self-worth.

^e^F5: compliance with ethics and roles.

^f^F6: nursing practice competence.

^g^F7: nurse-client relationship.

^h^F8: nursing sensitivity.

#### Factor 1

Named “Client-Centered Collaborative Practice,” this factor included 11 items. The eigenvalue was 17.79, explaining 12.1% of the variance, with factor loadings ranging from 0.45 to 0.72.

#### Factor 2

Named “Learning Agility for Nursing,” this factor included 4 items. The eigenvalue was 2.55, explaining 20.5% of the variance, with factor loadings ranging from 0.54 to 0.81.

#### Factor 3

Named “Nursing Professional Commitment,” this factor included 4 items. The eigenvalue was 2.12, explaining 28.2% of the variance, with factor loadings ranging from 0.54 to 0.82.

#### Factor 4

Named “Positive Self-Worth,” this factor included 5 items. The eigenvalue was 1.83, explaining 35.3% of the variance, with factor loadings ranging from 0.52 to 0.72.

#### Factor 5

Named “Compliance with Ethics and Roles,” this factor included 5 items. The eigenvalue was 1.50, explaining 42.2% of the variance, with factor loadings ranging from 0.52 to 0.71.

#### Factor 6

Named “Nursing Practice Competence,” this factor included 3 items. The eigenvalue was 1.45, explaining 48.6% of the variance, with factor loadings ranging from 0.76 to 0.80.

#### Factor 7

Named “Nurse-Client Relationship,” this factor included 3 items. The eigenvalue was 1.22, explaining 55.1% of the variance, with factor loadings ranging from 0.63 to 0.75.

#### Factor 8

Named “Nursing Sensitivity,” this factor included 3 items. The eigenvalue was 1.15, explaining 60.4% of the variance, with factor loadings ranging from 0.58 to 0.65.

### Confirmatory Factor Analysis

To validate the model structure of the eightfold 8 factors and 38 items derived from the EFA, CFA was conducted using data from 256 participants who were not included in the exploratory analysis. The model fit indices were *χ*²/df=2.48 (less than 3), RMSEA=0.07 (less than 0.10), SRMR=0.04 (less than 0.08), GFI=0.92 (greater than 0.90), and CFI=0.92 (greater than 0.90), all of which indicated an acceptable fit (Table 3).

**Table 3:** Result of confirmatory factor analysis.

	*χ*^2^ (*df*)	*P* value	*χ*^2^/*df*	RMSEA^a^	SRMR^b^	GFI^c^	CFI^d^	β	CR^e^	AVE^f^
ETNCM^g^	573.3 (198)	<.001	2.5	0.07	0.04	0.92	0.92	.60-.85	0.82-0.94	0.62-0.78
Criteria	—^h^	>.05	≤3	≤0.10	≤0.05	≥0.90	≥0.90	≥0.50	≥0.50	≥0.50

^a^RMSEA: root mean square error of approximation.

^b^SRMR: squared root mean-squared residual.

^c^GFI: goodness of fit index.

^d^CFI: comparative fit index.

^e^CR: construct reliability.

^f^AVE: average variance extracted.

^g^ETNCM: Emotional Touch Nursing Competencies Model.

^h^Not applicable.

### Convergent Validity

The convergent validity was confirmed, with standardized regression coefficients ranging from 0.60 to 0.85, all exceeding 0.50 (*P*<.05). The construct reliability ranged from 0.82 to 0.94, all exceeding 0.50, and the AVE ranged from 0.62 to 0.78, all exceeding 0.50. The discriminant validity of the factor structure was confirmed by verifying that the squared values of the correlations between latent variables were smaller than the AVE values in all areas (Table 3).

### Criterion Validity Verification

To verify the criterion validity of the developed emotional touch nursing competencies model, the Human-Centered Nursing Tool [23] was used. The correlation between Human-Centered Nursing and emotional touch nursing competencies was positively significant (*r*=0.78; *P*<.001). The correlations between subfactors of emotional touch nursing competencies and Human-Centered Nursing were also significant—client-centered collaborative practice (*r*=0.76; *P*<.001), nursing learning agility (*r*=0.48; *P*<.001), nursing professional commitment (*r*=0.49; *P*<.001), positive self-worth (*r*=0.58; *P*<.001), compliance with ethical and role (*r*=0.66; *P*<.001), nursing practice ability (*r*=0.46; *P*<.001), patient relationship ability (*r*=0.57; *P*<.001), and nursing sensitivity (*r*=0.55; *P*<.001), thereby confirming the criterion validity (Table 4).

**Table 4:** Correlation of the Emotional Touch Nursing Competencies Model (ETNCM) and Person-Centered Care Assessment Tool (P-CAT).

		F1^a^		F2^b^	F3^c^		F4^d^		F5^e^		F6^f^		F7^g^		F8^h^		ETNCM
**P-CAT**																	
	*r*	0.76	0.48		0.49	0.58		0.66		0.46		0.57		0.55		0.78	
	*P* value	<.001	<.001		<.001	<.001		<.001		<.001		<.001		<.001		<.001	

^a^F1: client-centered collaborative practice.

^b^F2: learning agility for nursing.

^c^F3: nursing professional commitment.

^d^F4: positive self-worth.

^e^F5: compliance with ethics and roles.

^f^F6: nursing practice competence.

^g^F7: nurse-client relationship.

^h^F8: nursing sensitivity.

### Reliability Verification

The internal consistency of the scale developed in this study was confirmed, with a Cronbach α of 0.95 for the overall scale. The reliability for each subfactor was as follows: factor 1=0.90, factor 2=0.84, factor 3=0.85, factor 4=0.83, factor 5=0.76, factor 6=0.86, factor 7=0.75, and factor 8=0.75.

### Optimization of the Scale and Finalization of the Competency Model

This study constructed an emotional touch nursing competencies model and verified its validity and reliability, resulting in a self-reported questionnaire consisting of 38 items across 8 factors, measured on a 5-point Likert scale. The total score ranges from 38 to 190, with higher scores indicating higher emotional touch nursing competencies.

**Figure 1:**
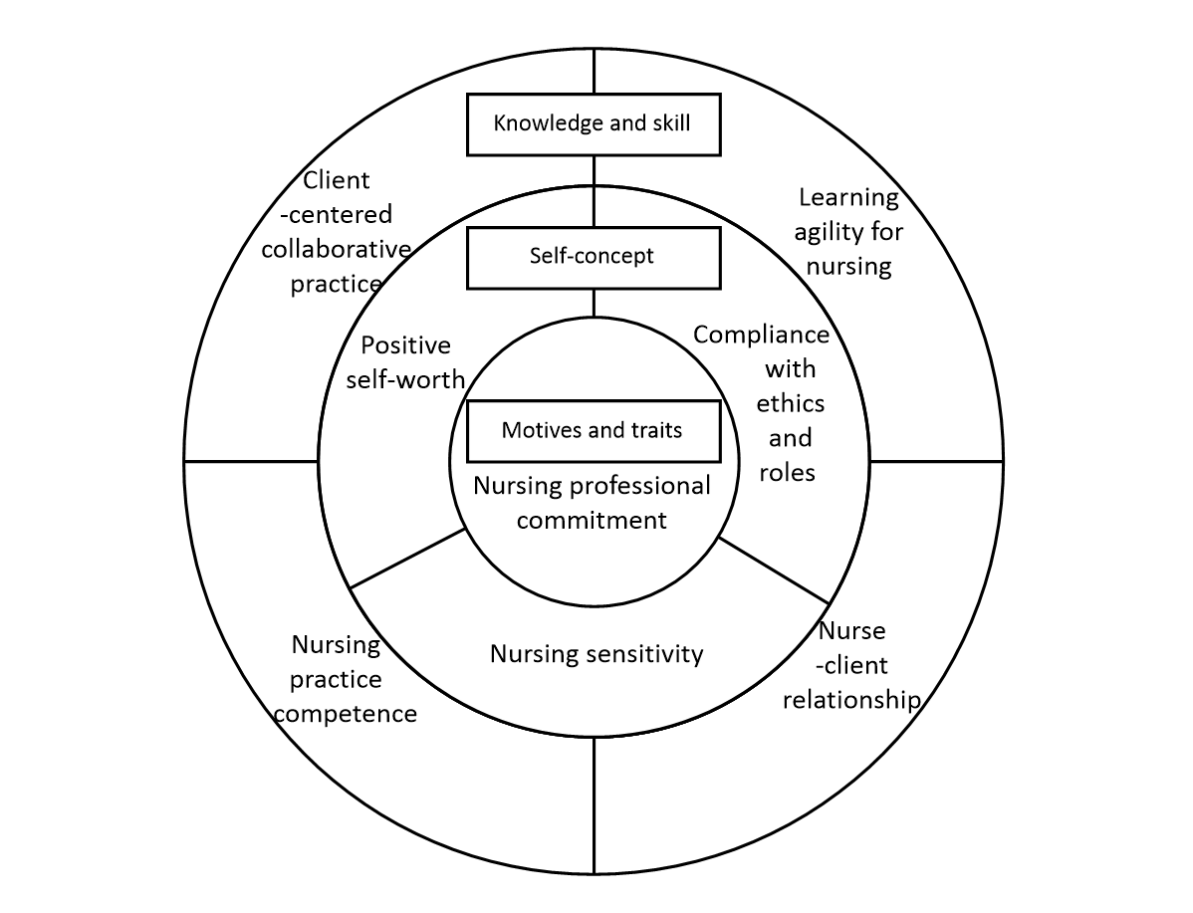
The competency model for emotional touch nursing.

The construct validity of the competency model was confirmed using the emotional touch nursing competencies scale centered on the Fourth Industrial Revolution, as defined by the competency model. Knowledge and skill competencies were finalized as client-centered collaborative practice, nursing learning agility, nursing practice ability, and nurse-client relationship ability. Self-concept competencies included positive self-worth, nursing sensitivity, and compliance with ethics and roles. Motivation and trait competencies were consolidated under nursing professional commitment (Figure 1).

## Discussion

### Principal Findings

This methodological study developed a scale for assessing emotional touch nursing competencies based on the concept and competency modeling of emotional touch nursing led by nurses in the Fourth Industrial Revolution. The developed scale consists of 38 items across 8 factors.

The competencies derived from this study include several key factors. The first factor, “Client-Centered Collaborative Practice,” consists of 11 items that encompass providing nursing care through collaboration in physical, psychological, environmental, and social aspects centered around the client. In light of the rapid advancements in advanced medical technologies and the increasingly complex and diverse competencies required of nurses to meet various client needs [24], this finding aligns with research by Seomun et al [25], which identified the integration of knowledge and nursing skills as essential core competencies for nurses. Additionally, client-centered care emphasizes the importance of client participation at all stages of health care [26]. Given these results, the factor of client-centered collaborative practice is deemed appropriate for providing diverse and nuanced nursing care, fostering trust in health care professionals amidst the rapidly evolving medical landscape.

The second factor, “Learning Agility for Nursing,” comprises 4 items related to the use of information and communication technologies and various data resources. This finding is consistent with the study by Kwak et al [27], which identified nursing informatics competency as a crucial skill related to nurses’ job performance. Furthermore, research by Lee et al [28] highlighted that the area with the lowest performance in nursing informatics subcompetencies pertains to the application of software related to medical information systems. These results reflect the learning vulnerabilities among clinical nurses in using software tools for patient care. As clinical nurses operate in environments deeply intertwined with rapidly advancing technologies in the Fourth Industrial Revolution, the importance of nursing learning agility is underscored for providing systematic, evidence-based nursing care.

The third factor, “Nursing Professional Commitment,” includes 4 items related to job pride and self-actualization. Job commitment, a concept similar to professional commitment, refers to the psychological pride, unity, and perceived importance one feels toward their current work [29]. Han and Koo’s [30] study found that professional self-concept was the most significant influencing factor on nurses’ job commitment, which aligns with our findings. The increase in job commitment stemming from a positive professional self-concept is anticipated to enhance the quality of nursing services, suggesting that when clinical nurses possess high pride, responsibility, and confidence in their profession, they can provide detailed and compassionate care to patients.

The fourth factor, “Positive Self-Worth,” encompasses 5 items related to personal values, self-efficacy, and self-esteem. Previous studies have demonstrated a significant negative correlation between positive psychological competencies and burnout [31]. Furthermore, nurses’ self-efficacy significantly influences patient safety management activities [32]. In this context, “positive self-worth” is seen as a critical concept for nurses in recognizing their positive value and establishing their self-concept, contributing to the growth of their emotional touch.

The fifth factor, “Compliance with Ethics and Roles,” consists of 5 items related to nursing ethics, professional role responsibilities and duties, and respect for clients. This finding supports previous studies that emphasized the importance of ethical competency in general nursing competencies [33]. The Singapore Nursing Board has suggested ethical, professional, and legal nursing practices; care management; leadership; and professional development as competencies for nurses [34], aligning with the results of this study.

The sixth factor, “Nursing Practice Competence,” includes 3 items related to various clinical nursing skills applicable to patients. This factor is comparable to clinical performance [35], clinical decision-making abilities [36], and job performance [27] identified in prior studies. However, unlike similar concepts in previous research, the nursing practice competency factor reflects the characteristics of the nursing profession while distinguishing itself through the provision of client-centered nursing care suitable for the Fourth Industrial Revolution. Delivering high-quality care based on nursing practice competency is an essential condition for nurses.

The seventh factor, “Nurse-Client Relationship,” comprises 3 items related to relationships, communication, and attitudes toward clients. This aligns with findings from prior studies [37] investigating the professional competencies of nurses in various departments, highlighting the importance of behaviors that foster a close connection between nurses and clients. Furthermore, research analyzing recent data from domestic and international accreditation agencies identified communication competency as a priority for improvement [25], indicating that interpersonal issues among nurses significantly affect the quality of nursing services [38]. Thus, enhancing client relationship competency is crucial for improving patient satisfaction with nursing services.

The eighth factor, “Nursing Sensitivity,” includes 3 items concerning respect for client treatment, empathy for vulnerable situations, and prompt responses. This finding is consistent with prior studies emphasizing the immediate provision of individualized patient care as a vital component of nurses’ professional competencies [37]. Moreover, recent patient expectations for high-quality nursing care have expanded to include an empathetic understanding of their situations and emotions, reflecting the evolving scope of nursing in a professional context [39].

Based on a literature review, interviews with nurses and nursing clients, and 3 rounds of expert Delphi and content validity verification processes, 13 attributes and 51 items were ultimately derived, with 50 items undergoing factor analysis to confirm the 8 factors comprising 38 items. Additionally, these 8 factors accounted for 60.43% of the total variance. The reliability of the scale was found to be Cronbach α=0.95, with subfactor reliabilities ranging from 0.75 to 0.90. Correlation analysis for criterion validity revealed a positive correlation between person-centered care [23] and emotional touch nursing competencies, with all subfactors also demonstrating positive correlations.

This study developed a scale to measure competencies defined based on the competency model to verify the construct validity of the competency model. Competency modeling for nursing integrates cognitive and noncognitive skills systematically and contextually based on internal structures [20]. Therefore, the results of this study approached from the integrated perspective of knowledge and skills, self-concept, motivation, and traits, provide valuable insights into the emotional touch nursing competencies of nurses.

This research is significant for attempting to build a model of emotional touch nursing competencies based on competency modeling, considering the rapidly changing nursing demands of clients in the Fourth Industrial Revolution and post–COVID-19 era. This scale can be used to assess and evaluate the degree of emotional touch competencies among nurses, providing a foundation and essential data for developing educational programs aimed at enhancing emotional touch nursing competencies.

### Conclusions

This study established a model of emotional touch nursing competencies for nurses in response to the Fourth Industrial Revolution through the modeling of the concept and competencies of emotional touch nursing. The model encompasses 8 factors—“Client-Centered Collaborative Practice,” “Learning Agility for Nursing,” “Nursing Practice Competence,” and “Nurse-Client Relationship,” which reflect knowledge and skills; “Positive Self-Worth,” “Compliance with Ethics and Roles,” and “Nursing Sensitivity,” which represent self-concept; and “Nursing Professional Commitment,” which pertains to motivation and traits. The scale developed for this study consists of a total of 38 items using a 5-point Likert scale, with a possible score range of 38 to 190, indicating that higher scores reflect higher emotional touch nursing competencies. This scale is expected to be useful for assessing nurses’ emotional touch nursing competencies and, through the results, for developing and implementing educational programs aimed at enhancing nursing competencies, thereby facilitating the provision of positive emotional touch nursing to clients in the rapidly changing health care environment of the Fourth Industrial Revolution.

Based on the findings of this study, the following suggestions are made—first, as this study targeted clinical nurses in general hospitals or larger institutions, future research is recommended to explore the degree of emotional touch nursing competencies among nurses in hospitals of various sizes. Second, subsequent research is suggested to develop and apply educational programs aimed at enhancing nurses’ emotional touch nursing competencies based on the model developed in this study.

## Abbreviations

AI: artificial intelligence
AVE: average variance extracted
CFA: confirmatory factor analysis
CFI: comparative fit index
EFA: exploratory factor analysis
GFI: goodness of fit index
I-CVI: Item-Content Validity Index
KMO: Kaiser-Meyer-Olkin
P-CAT: Person-Centered Care Assessment Tool
RMSEA: root mean square error of approximation
SRMR: squared root mean-squared residual
